# Effects of Polysaccharide Supplementation on Lactic Acid Bacteria-Fermented Soy Protein Gel: Structural Characteristics, Allergenicity, and Epitope Analysis

**DOI:** 10.3390/foods14040701

**Published:** 2025-02-18

**Authors:** Xinran Guo, Yun Luo, Xia Fan, Benjamin K. Simpson, Wei Li, Xin Rui

**Affiliations:** 1 College of Food Science and Technology, Nanjing Agricultural University, Nanjing 210095, China; guo.daisy.m@outlook.com (X.G.); luo752701@163.com (Y.L.); fanxia@njau.edu.cn (X.F.); lw1981@njau.edu.cn (W.L.); 2 Department of Food Science and Agricultural Chemistry, McGill University, Montreal, QC H9X 3V9, Canada; benjamin.simpson@mcgill.ca

**Keywords:** soy protein isolate, lactic acid bacteria fermentation, polysaccharide, texture properties, in vitro dynamic gastrointestinal digestion, antigenicity, IgE-binding capacity, peptidomic

## Abstract

Background: Soy allergy is an important nutritional and health issue that needs to be addressed. Lactic acid bacteria (LAB) fermentation is an effective approach to reduce soy protein allergy. Polysaccharides are commonly used in LAB-fermented products to enhance their textural properties. This study proposes a new strategy for developing hypoallergenic soy protein products. Methods: We prepared a soy protein isolate (SPI) through fermentation with LAB (FSPI) and with five types of polysaccharides supplementation, namely polydextrose (PDX), inulin (IN), long-chain inulin (LCIN), soluble soy polysaccharides (SSPS), and β-glucan (BG). The texture and microstructure of different samples were analyzed. Antigenicity and IgE-binding capacity were determined using ELISA. Finally, peptide sequencing was used to identify the degradation degree and frequency of allergenic epitopes. Results: Samples with added PDX (F-PDX) and IN (F-IN) exhibited lower hardness; smaller, irregular pores; and a honeycomb structure, whereas samples with SSPS (F-SSPS) and BG (F-BG) had higher hardness; large, regular pores; and strong sheet structures. The antigenicity and IgE-binding capacity of F-PDX and F-IN were lower both before and after 120 min of in vitro dynamic gastrointestinal digestion. The peptidomics results indicated that F-PDX and F-IN primarily facilitated the degradation of the glycinin G1 and G2 subunits, β-conglycinin α, and the β subunit. Moreover, it increased the frequency of destruction of allergenic epitopes, and further promoted the degradation of epitopes in the external α-helix structures of glycinin and β-conglycinin compared to FSPI. Conclusions: The addition of polysaccharides had a significant impact on the structure and allergenicity of the soy protein gel, especially PDX and IN.

## 1. Introduction

Due to environmental, sustainability, and public health concerns, plant-based foods have garnered significant attention in recent years [1]. Plant proteins, such as soy and pea proteins, are increasingly becoming popular alternatives to animal-based meat, poultry, eggs, and dairy proteins. This shift is particularly significant in both the food industry and scientific research sectors [2]. Soy protein is a widely used protein with numerous nutritional and health benefits [3,4]. However, the potential allergenicity of soy protein poses a challenge, somewhat limiting the advancement and application of soy protein products [5]. Soy allergy is a significant nutritional and health concern, particularly for children and some adults, as allergic reactions may affect their dietary choices and nutrient intake [6]. Therefore, reducing soy allergenicity has an important impact on nutritional health.

Microbial fermentation presents a promising strategy for mitigating the allergenicity of soy proteins [7]. Fermentation can alter protein structures and degrade allergenic proteins, thereby reducing their allergenic potential [8]. Previous studies have demonstrated that lactic acid bacteria (LAB) fermentation facilitates the degradation of allergenic epitope sequences in soy protein [9]. Another study indicated that LAB fermentation leads to a reduction in α-helices and an increase in β-sheets within the soy protein structure, alongside a decrease in hydrogen bonds, which consequently lowers allergenicity [10].

LAB fermentation generally leads to the reorganization of soy protein, promoting gel formation under acidic conditions [11]. Studies on acid-induced soy protein gels have shown that they exhibit lower water retention and hardness, coupled with a soft texture and a loose, open gel network [12]. The incorporation of thickeners, such as polysaccharides, can address the limitations associated with gel characteristics. Polysaccharides are widely found in nature and possess various biological activities and essential functions [13]. In the food industry, polysaccharides are commonly utilized as thickeners, stabilizers, and gelling agents. Soluble soy polysaccharides (SSPS) and inulin (IN), for example, have been shown to interact with proteins, thereby modifying their physicochemical and textural properties. Previous studies have explored the emulsifying characteristics of non-covalent complexes of soy protein and SSPS, demonstrating that the addition of polysaccharides significantly enhances the emulsifying activity and stability of soy protein [14]. Another study examined the effects of IN on yogurt, indicating that increased IN levels improved its firmness and creaminess [15].

Although some preliminary studies have explored the effects of polysaccharides on the structure of soy protein, research on their impact on the allergenicity of soy protein remains relatively limited. Based on this, the hypothesis of this study is that during the fermentation of soy protein with LAB, the addition of polysaccharides may reduce allergenicity by altering the structure of soy protein and its immune response characteristics. Therefore, this study aimed to prepare soy protein gels through fermentation with LAB, both in the presence and absence of five types of polysaccharides: polydextrose (PDX), IN, long-chain inulin (LCIN), SSPS, and β-glucan (BG). The influence of these polysaccharides on the textural and immunoreactive characteristics of fermented soy protein isolate (FSPI) was examined, and immunoreactivity changes were tracked using in vitro dynamic gastrointestinal digestion experiments. Additionally, peptidomic and immunoinformatic analyses were used to study the disruption profiles of allergenic epitopes following the addition of various polysaccharides. This study proposes reducing the allergenicity of soy protein through lactic acid bacteria fermentation and polysaccharide supplementation, making soy protein a safer food option for individuals with allergies and enhancing its nutritional acceptability and applicability.

## 2. Materials and Methods

### 2.1. Materials, Chemicals, and Microorganisms

All soybeans were sourced from a soybean base in Northeast China. The *Lactobacillus plantarum* B1-6 strain (the gene accession number was KM200717) used in this research was originally isolated from traditional fermented grains and beverages in Xinjiang Uyghur Autonomous Region, China. RIDASCREEN^®^FAST-Soya were purchased from R-Biopharm AG (Darmstadt, Germany). All the other chemical reagents used were of analytical grades.

### 2.2. Preparation of Soy Protein Isolate (SPI)

**Figure 1:**
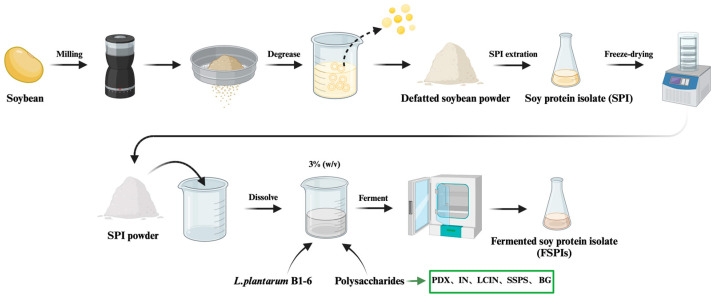
Preparation of SPI and FSPIs.

The extraction of SPI was conducted using a method adapted from L’hocine et al. [16]. First, the soybeans were ground three times using a grinder (RS-FS1401, Royalstar, Hefei, China) with a rotational speed set at 6000–10,000 rpm, followed by sieving through a 40-mesh standard sieve. Soybean flour and hexane were mixed at a 1:3 *w*/*v* ratio and stirred at room temperature for 45 min, followed by centrifugation (5000× *g*, 15 min, 25 °C). The precipitate was collected, and the process was repeated three times to obtain defatted soybean flour. The defatted powder was mixed with 15-fold distilled water, with the pH adjusted to 9.0, and stirred at 45 °C for 1 h. Following this, the mixture was centrifuged for 15 min at 10,000× *g* and 4 °C to isolate the supernatant. The pH of the supernatant was then adjusted to 4.5 and stirred at 25 °C for 1 h. The mixture underwent a second centrifugation under the same conditions, and the resultant precipitate was freeze-dried to obtain SPI (Figure 1).

### 2.3. Preparation of Six Fermented SPI Gels (FSPIs)

The *L. plantarum* B1-6 strain was cultured twice in Man–Rogosa–Sharpe (MRS) broth. Following cultivation, the cells were centrifuged for 15 min at 4000× *g* and washed three times with physiological saline. The freeze-dried SPI powder was dissolved in water, adjusted to pH 7.0, and pasteurized at 85 °C for 10 min. After pasteurization, a sterile glucose solution was added to achieve a final concentration of 2.0% (*w*/*v*), and the suspension was inoculated with *L. plantarum* B1-6 at a final concentration of 3.0% (*w*/*v*). Fermentation was conducted both without polysaccharides and with different polysaccharides (at a final concentration of 2.0% (*w*/*v*)), namely PDX, IN, LCIN, SSPS, and BG, respectively, resulting in the following gel samples: FSPI, F-PDX, F-IN, F-LCIN, F-SSPS, and F-BG (Figure 1). The chemical properties of different polysaccharides are shown in Table 1. The fermentation process was carried out at 37 °C, with a target pH of 5.0 established as the endpoint for all tests. The time required to reach pH 5.0 varied among samples, ranging from 10 to 16 h.

### 2.4. Texture Property

The hardness, springiness, cohesiveness, and gumminess were evaluated using a TMS-Pro texture analyzer (Fairfax, VA, USA). A cylindrical flat-headed probe with a 2 cm diameter (p/50 model) was used for the measurements. The entry and testing speeds were set to 1 mm/s and 5 mm/s, respectively, with a trigger force of 5 g, compressing the sample by 30%. Each measurement was performed in triplicate with two parallel sets per sample.

### 2.5. Microstructure Analysis

A Hitachi SU8010 cryo-electron microscope (Hitachi, Tokyo, Japan) was used to examine the microstructure of each FSPI. Five microliters (5 µL) of each fermented sample were placed in a specialized tank, with the temperature rapidly lowered to liquid nitrogen temperatures (−196 °C) using ultra-fast cooling technology at 15 kV. The frozen samples were analyzed under vacuum to observe their internal structure.

### 2.6. In Vitro Dynamic Gastrointestinal Digestion

Simulated gastric fluid (SGF) and simulated intestinal fluid (SIF) were prepared according to the methodology outlined by Minekus et al. [17]. The SGF consisted of porcine gastric mucosa and gastric mucin dissolved in a buffer containing NaHCO_3_, NaCl, KCl, KH_2_PO_4_, MgCl_2_(H_2_O)_6_, and (NH_4_)_2_CO_3_, with the pH adjusted to 1.6. For the preparation of SIF, pancreatin and bile salts were dissolved in a buffer containing NaHCO_3_, NaCl, KCl, KH_2_PO_4_, and MgCl_2_(H_2_O)_6_, maintaining a pH of 7.0. Both SGF and SIF were then heated in a water bath at 37 °C for 20 min to activate the enzymes.

In vitro dynamic gastrointestinal digestion was performed using the Bionic Rat Model II+ (Xiao Dong Pro-health Instrumentation Co., Ltd., Suzhou, China). SGF and SIF were injected at 52 μL/min. The device replicated gastric movements with a compression frequency of 3 cycles per minute (cpm) and a rolling frequency of 12 cpm, whereas the intestinal rolling frequency was set to 36 cpm. Digested samples (1 mL each) were collected at 30, 60, 90, 120, 150, and 180 min, designated as I-30, I-60, I-90, I-120, I-150, and I-180, respectively. Each sample, including the control samples at time zero, was immediately subjected to a 5 min boiling water bath to deactivate the enzymes, then stored at −80 °C.

### 2.7. Allergenicity Assays

#### 2.7.1. Antigenicity Analysis

Antigenicity was assessed using the RIDASCREEN^®^FAST Soya ELISA kit (R7102, R-Biopharm AG, Darmstadt, Germany), following the protocol established by L’hocine and Minekus [16,17]. First, 100 µL of diluted samples or standards were added to each well of a microtitration plate pre-coated with a primary antibody and incubated at 25 °C for 10 min. The wells were then washed three times. Subsequently, 100 µL of secondary antibody was added, and the plate was incubated again at 25 °C for 10 min. The washing process was repeated, after which 100 µL of substrate/color developer was introduced. To protect the plate from light, it was covered with tin foil and placed in the dark for 10 min. To ensure a complete reaction, 100 µL of stop solution was added. The absorbance was measured at 450 nm. Then, the antigenicity of the protein samples was calculated based on the standard curve generated from the standard samples and was calculated using Formula (1). The control group refers to the antigenicity of the unfermented sample (SPI). The standard curve and absorbance for each sample was provided in the Supplementary Materials (Figure S1 and Table S1). All experiments were performed in triplicate.

(1) $$\mathrm{Antigenicity}\;(\%)=\frac{\mathrm{Concentration}\;\left(\mathrm{samples}\right)}{\mathrm{Concentration}\left(\mathrm{control}\right)}\;\times \;100$$

#### 2.7.2. IgE-Binding Capacity Analysis

As described by previous studies [18,19,20], the enzyme-linked immunosorbent assay (ELISA) was carried out using human serum with soy allergens. Serum from eight patients with documented soy allergy reactions was obtained from Wolcavi Biotechnology Co., Ltd. (Chongqing, China). These patients ranged in age from 22 to 65 years and had specific IgE levels ranging from 2.43 to 8.43 kU/L. Protein samples were prepared by dilution in a sodium carbonate buffer (10 mmol, pH 9.5), and 100 μL of each sample was added into the wells. The plate was incubated for 20–24 h, then washed 3–5 times with the PBS buffer with 0.05% Tween-20 (*v*/*v*), with each wash followed by being patted dry. After washing, 250 μL of a blocking solution was introduced, and the samples were incubated at 37 °C for 1.5 to 2.5 h. Following an additional 3–5 washes, 100 μL of human serum diluted in PBS with 1% (*v*/*v*) BSA was added, and the plate was incubated at 37 °C for 1 h. The wells were then washed again, and 100 μL of anti-human IgE (ε-chain specific)-peroxidase antibody diluted in PBS was added. The plate underwent another incubation at 37 °C for 1 h, followed by three washes. Finally, 100 μL of 3,3′,5,5′-Tetramethylbenzidine (TMB) was added, and the plate was incubated in the dark at 37 °C for 10 min, after which the reaction was halted by adding 100 μL of H_2_SO_4_. The absorbance was measured at 450 nm, and the entire experiment was conducted in triplicate. The IgE-binding capacity was calculated using Formula (2). The control group refers to the IgE-binding capacity of the unfermented sample (SPI). All experiments were performed in triplicate.

(2) $$\mathrm{IgE}\text{-}\mathrm{binding}\;\mathrm{capacity}\;(\%)=\frac{{\mathrm{OD}}_{450}\left(\mathrm{samples}\right)}{{\mathrm{OD}}_{450}\left(\mathrm{control}\right)}\;\times \;100$$

### 2.8. Peptidomic Identification

Peptide identification in the protein samples was performed using liquid chromatography–mass spectrometry (LC-MS). A 10% (*w*/*w*) solution of trichloroacetic acid was initially added and allowed to incubate for 5 h. The samples were then centrifuged at 6000× *g* for 15 min to precipitate the peptides. Subsequently, the peptides were desalted using Pierce™ C18 spin columns (Thermo Fisher Scientific, Mississauga, ON, Canada). After desalination, the vacuum dried peptide was resuspended in 0.1% formic acid (FA) and the concentration was adjusted to 0.5 μg/μL. For the analysis, 2 μL of the peptide solution was loaded into an online nano LC system (EASY-nLC 1200, Thermo Scientific) connected to a mass spectrometer. Peptides were separated using an Acclaim PepMap^®^ RSLC, C18, 75 μm × 15 cm, 3 μm, 100Å analytical column. The mass spectrometer operated in data-dependent analysis (DDA) mode. Raw data from the mass spectrometry analysis were processed using PEAKS Studio software v.12.5 (Bioinformatics Solutions, Waterloo, ON, Canada) against a soybean protein database sourced from UniprotKB (https://www.uniprot.org). The search parameters were established with a false discovery rate (FDR) of ≤1% and an average local confidence (ALC) of ≥99%, based on de novo sequencing.

### 2.9. Bioinformatics Analysis of Epitope Degradation

Data extracted from the Immune Epitope Database (IEDB) revealed the number of allergenic epitopes present in various soybean protein subunits. The amino acid sequences of these allergenic proteins were obtained from UniProtKB (https://www.uniprot.org). Using Chimera software, version 1.15 (UCSF, USA), three-dimensional structural models of these proteins were constructed. The degradation rates and frequencies of allergenic epitopes were analyzed based on the peptidomics results, with the degradation rate of the allergenic epitopes determined using the Formula (3):

(3) $$\mathrm{Allergenic}\;\mathrm{epitope}\;\mathrm{degradation}\;\mathrm{rate}\;(\%)=\frac{\mathrm{Number}\;\mathrm{of}\;\mathrm{allergenic}\;\mathrm{epitopes}\;\mathrm{disrupted}}{\mathrm{Total}\;\mathrm{allergenic}\;\mathrm{epitopes}}\;\times \;100$$

### 2.10. Statistical Analysis

Data from the experiments were analyzed utilizing Statistical Product and Service Solutions (SPSS) software, version 29.0.1.0 (SPSS, Inc., Chicago, IL, USA) to perform a one-way ANOVA. Results from three replicate experiments were averaged, and statistical significance was assessed using Duncan’s test (*p* < 0.05). Graphs and charts were constructed using GraphPad Prism 10.0 (GraphPad Software, Inc., San Diego, CA, USA).

## 3. Results and Discussion

### 3.1. Textural Features of FSPIs

The effects of different types of polysaccharides on the texture of FSPIs are presented in Table 2. The hardness of F-PDX and F-IN (0.27–0.28 N) was similar to that of FSPI, but significantly lower than that of F-LCIN, F-SSPS, and F-BG (0.36–0.39 N) (*p* < 0.05). This suggests that the addition of PDX and IN contributes to a softer gel structure. The springiness of FSPIs with added polysaccharides was significantly improved compared to FSPI (*p* < 0.05), with the following ranking from highest to lowest: F-LCIN > F-SSPS > F-PDX, F-IN, F-BG > FSPI. All FSPIs exhibited similar cohesiveness. Except for F-PDX, the gumminess of the other samples was comparable.

### 3.2. Microstructure

**Figure 2:**
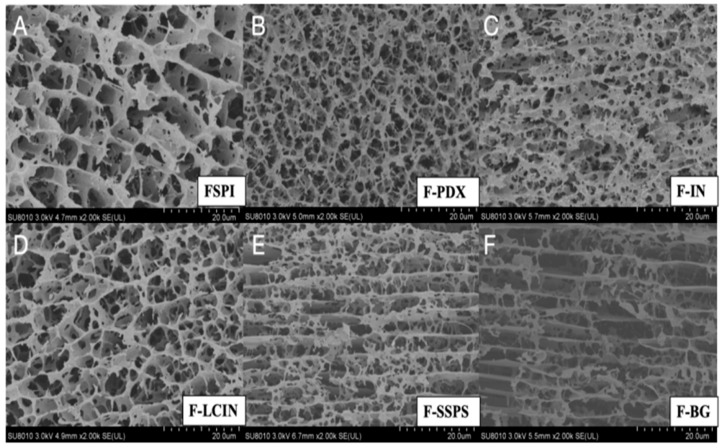
Cryo-SEM images of FSPIs through fermentation with LAB in the presence and absence of five types of polysaccharides. (**A**) FSPI, (**B**) F-PDX, (**C**) F-IN, (**D**) F-LCIN, (**E**) F-SSPS, and (**F**) F-BG.

Figure 2 presents cryo-SEM electron microscopy images of FSPI and five FSPIs with added polysaccharides. All FSPIs displayed a honeycomb three-dimensional network structure. FSPI (Figure 2A) evidenced an irregular lamellar honeycomb structure with large, heterogeneous pores of approximately 10 μm, which is consistent with previous studies [21,22]. F-PDX and F-IN revealed a disorganized, dense, and fragile filamentous network with smaller pores (~2 μm) (Figure 2B,C). F-LCIN had slightly smaller pores than FSPI (~8 μm), but maintained a similar sheet structure (Figure 2D). F-SSPS and F-BG exhibited more pronounced sheet structures with larger, regular pores (~15 μm) (Figure 2E,F). These findings suggest that different types of polysaccharides impact the structure of soy protein gels in distinct manners. Samples with lower hardness (F-PDX and F-IN) presented small, irregular pores and weaker honeycomb networks, whereas samples with higher hardness (F-SSPS and F-BG) showed large, regular pores and robust sheet structures. The structural changes in FSPI gels following the addition of LCIN were minimal.

### 3.3. Antigenicity and IgE-Binding Capacity Analysis

#### 3.3.1. Antigenicity

**Figure 3:**
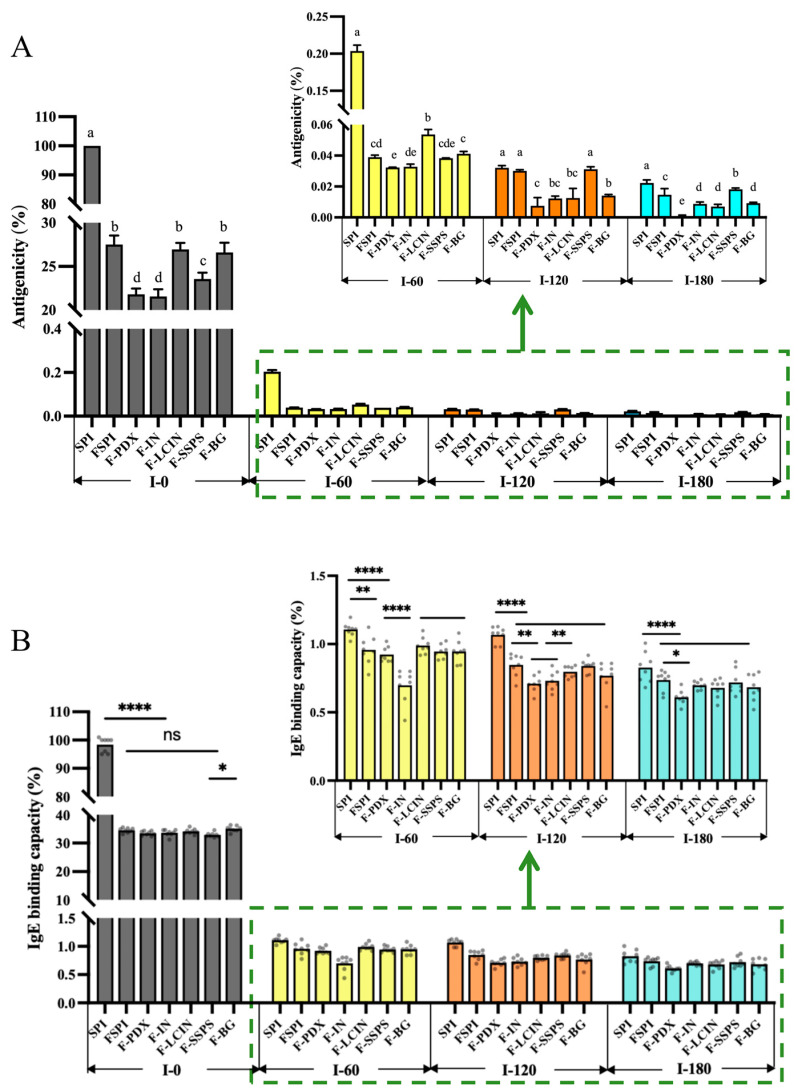
Antigenicity (**A**) and IgE-binding capacity (**B**) of the SPI digestate and FSPIs digestates collected during in vitro dynamic gastrointestinal digestion. The significant difference (*p* < 0.05) between the samples collected at the same time is denoted by lowercase letters (a–e). Analysis of variance level, * *p* < 0.05, ** *p* < 0.01, **** *p* < 0.0001.

Compared to SPI, the antigenicity of all FSPIs was reduced to below 30% (Figure 3A), indicating that the fermentation process significantly decreased antigenicity. Among these FSPIs, F-PDX and F-IN exhibited the lowest antigenicity, ranging from 21.56% to 21.79%, followed by F-SSPS (23.57%). The antigenicity of F-LCIN and F-BG was slightly higher (26.6–26.9%), and did not differ significantly from that of FSPI (27.5%) (*p* > 0.05). The samples were then tested in Bionic Rat Model II+. As digestion progressed, the antigenicity of all samples continued to decrease. At I-60, antigenicity significantly dropped to between 0.0323% and 0.2035%, with the SPI digestate remaining approximately seven times higher (0.2035%) than that of any FSPIs digestates (0.0323–0.0537%) (*p* < 0.05). Among all FSPIs, the F-PDX digestate showed the lowest antigenicity, whereas the F-LCIN digestate had the highest. The antigenicity differences between the other three polysaccharide samples and the FSPI digestate were not statistically significant (*p* > 0.05). At I-120, although the antigenicity of the SPI digestate decreased significantly, it remained notably higher than that of the F-PDX, F-IN, F-LCIN, and F-BG digestates (*p* < 0.05), with the F-PDX digestate remaining the lowest at 0.0075%. By the endpoint I-180, the antigenicity of the FSPI digestate (0.0004–0.0191%) was significantly lower than that of the SPI digestate (0.0223%) (*p* < 0.05). Except for the F-SSPS digestate, the antigenicity of digestates from other polysaccharide-added samples approached 0%, significantly lower than that of the FSPI digestate (*p* < 0.05).

#### 3.3.2. IgE-Binding Capacity

In comparison to SPI, all FSPIs showed a significant decrease in IgE-binding capacity, ranging from 32.88% to 34.41% (*p* < 0.05) (Figure 3B), indicating the effectiveness of fermentation in reducing immunoreactivity. FSPIs supplemented with polysaccharides exhibited lower IgE-binding capacities. F-SSPS showed the lowest value (32.88% ± 0.91%), and there was no significant difference when compared to F-PDX, F-IN, and F-LCIN (33.47–34.41%) (*p* > 0.05), suggesting that the addition of the polysaccharides SSPS, PDX, IN, and LCIN effectively mitigates sensitization.

Throughout the entire gastrointestinal digestion period (0–180 min), the IgE-binding capacity of the SPI digestate consistently remained higher than that of FSPIs digestates, with all values gradually decreasing over time. Among the FSPIs digestates, the samples supplemented with polysaccharide IN exhibited the lowest IgE-binding capacity at I-60 (0.70%), which was significantly lower than that of the FSPI digestate (0.96%) (*p* < 0.05). The remaining samples showed no significant differences from the FSPI digestate (*p* > 0.05). At I-120, the IgE binding capacities of the F-PDX, F-IN, and F-BG digestates decreased to 0.71–0.77%, which were significantly lower than those of the FSPI digestate and the digestates supplemented with LCIN and SSPS (0.80–0.85%) (*p* < 0.05). At I-180, the F-PDX digestate maintained the lowest IgE-binding capacity (0.61%). These results indicate that FSPIs digestates supplemented with polysaccharides exhibited relatively lower IgE-binding capacities, although the specific polysaccharide type plays a role.

This suggests that softer gels with small and irregular pores (such as F-PDX and F-IN) may facilitate greater enzyme penetration due to their more open or fragmented network, thereby enhancing the digestion of allergenic proteins and more effectively reducing allergenicity.

### 3.4. Peptidomics of SPI, FSPIs and Their Digestive Products

Among all FSPIs with added polysaccharides, F-PDX displayed the lowest antigenicity and IgE-binding capacity at I-120, followed closely by F-IN. Consequently, peptidomic methods were employed to compare the peptide profiles of SPI, FSPI, F-PDX, and F-IN at I-0 and I-120, with the aim of identifying major allergens and degraded epitopes.

#### 3.4.1. Epitope Degradation Rate of Soy Allergens

**Figure 4:**
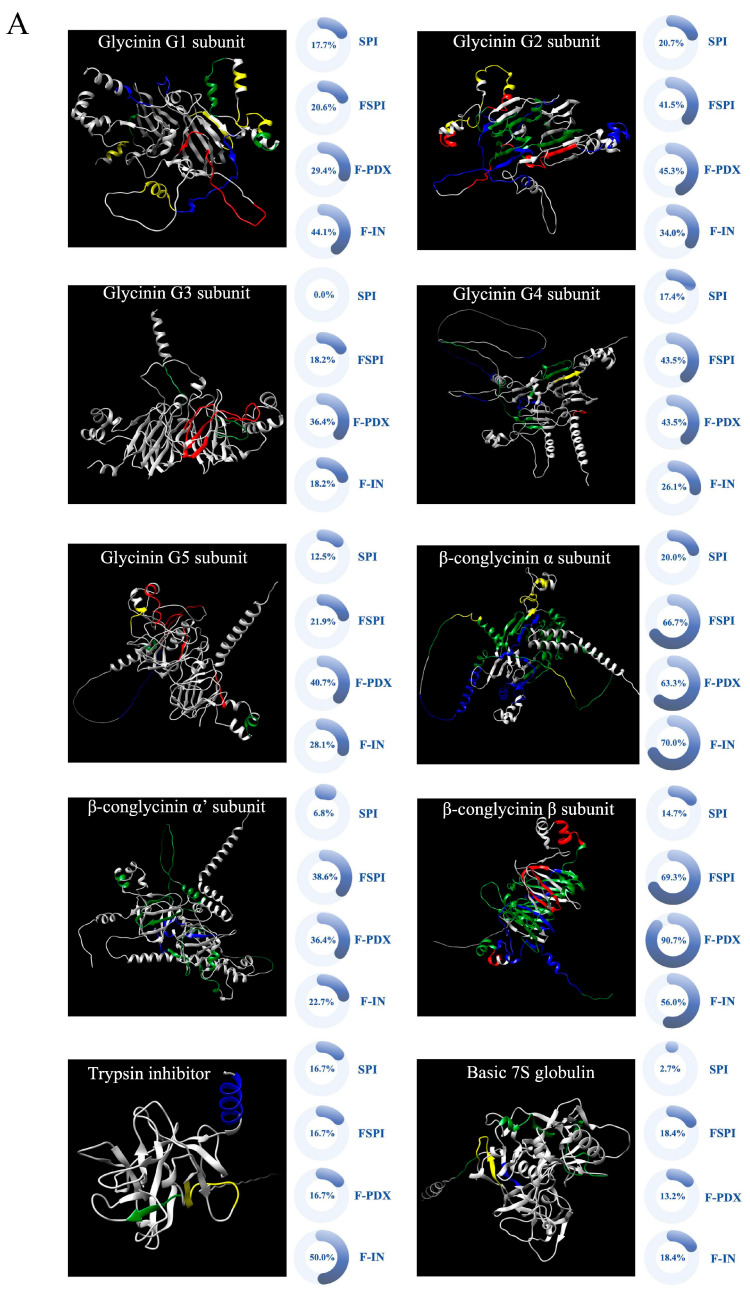

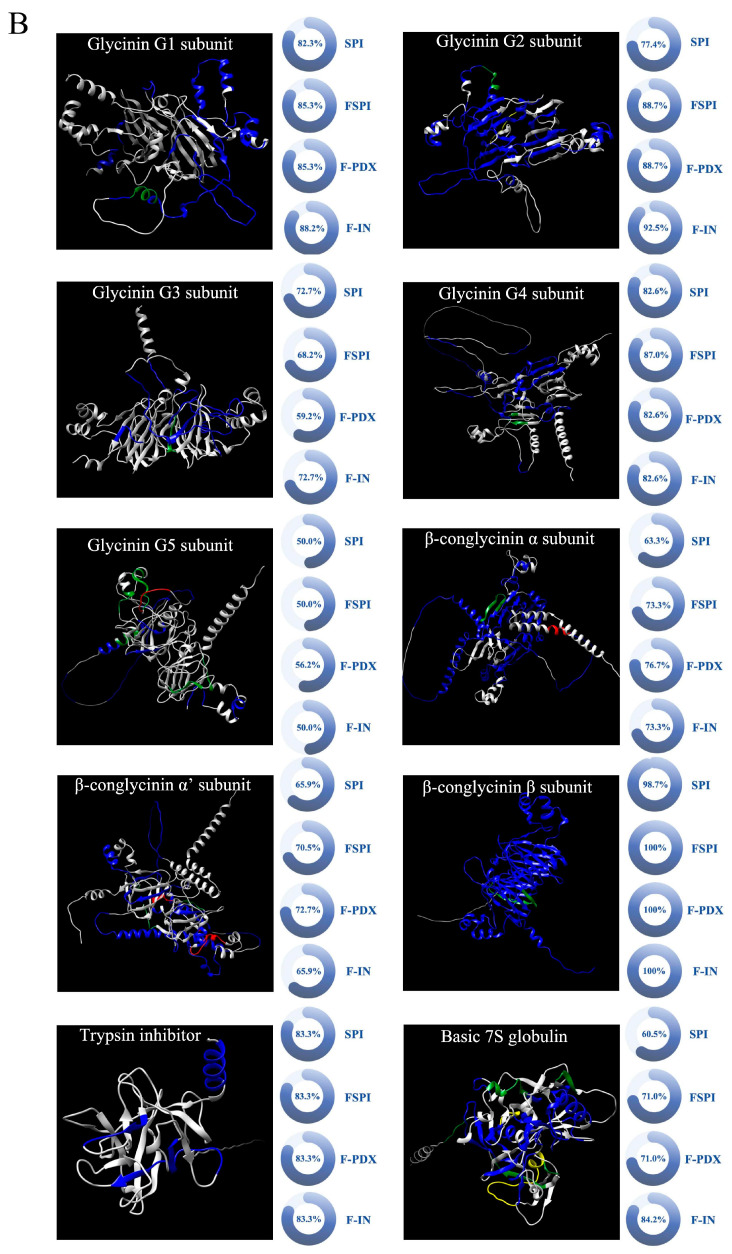
Epitope degradation rates of major soybean allergens and disrupted epitopes in 3D models at I-0 (**A**) and I-120 (**B**). (Disrupted allergenic epitopes in 3D models for SPI are marked with blue, whereas the additional epitopes that were further degraded in FSPI are marked with green, and the additional epitopes that degraded more than FSPI in F-PDX and F-IN are marked with red and yellow color).

Figure 4 illustrates the degradation rates of major soy allergen epitopes in samples before and after gastrointestinal digestion. Soy protein predominantly consists of 10 allergenic proteins. Compared to SPI, the three FSPIs showed higher epitope degradation rates, indicating that LAB fermentation promoted the degradation of allergenic epitopes, leading to a decrease in allergenicity. This discovery was consistent with the results of protein antigenicity analysis. Following gastrointestinal digestion, all samples exhibited significantly enhanced epitope degradation rates, particularly the two samples with added polysaccharides (F-PDX and F-IN).

Fermentation significantly increased the degradation rates of allergenic epitopes in soybean proteins (Figure 4A). This was especially evident in the glycinin G2, G3, and G4 subunits, as well as all β-conglycinin subunits and the basic 7S globulin. The degradation rates rose from 20.7%, 0.0%, 17.4%, 20.0%, 6.8%, 14.7%, and 2.7% for SPI to 41.5%, 18.2%, 43.5%, 66.7%, 38.6%, 69.3%, and 18.4% for FSPI, respectively. The 3D structural diagrams show that FSPI enhanced the degradation of all soy allergens (highlighted in green, Figure 4A). Previous research has shown that fermentation promotes the breakdown of allergenic epitopes. Once degraded, these epitopes may no longer bind to specific immunoglobulin E (IgE), reducing their allergenic potential [23]. Our findings support this and show that adding polysaccharides to the soy fermentation mixture further boosts the degradation of allergenic epitopes, particularly in the glycinin subunits and α and β subunits of β-conglycinin, with the degraded epitopes predominantly located in the protein’s external α-helix structures (highlighted in red and yellow, Figure 4A). Among the FSPIs, samples with added polysaccharide PDX had higher degradation rates for the G1, G2, G3, and G5 subunits of glycinin and the β subunit of β-conglycinin. Degradation rates increased from 20.6%, 41.5%, 18.2%, 21.9%, and 69.3% in FSPI to 29.4%, 45.3%, 36.4%, 40.7%, and 90.7% in F-PDX (highlighted in red, Figure 4A). Structural diagrams show that the further degraded epitopes in PDX-incorporated FSPI are concentrated in the irregular curls and α-helix regions on the protein’s exterior. In contrast, samples with added polysaccharide IN showed enhanced degradation for the G1 and G5 subunits of glycinin, the α subunit of β-conglycinin, and the trypsin inhibitor. Degradation rates increased from 20.6%, 21.9%, 66.7%, and 16.7% in FSPI to 44.1%, 28.1%, 70.0%, and 50.0% in F-IN (highlighted in yellow, Figure 4A). The structural diagrams show that F-IN further enhanced the degradation of the external α-helix structures of these protein subunits compared to FSPI.

At I-120, all samples exhibited a significant increase in the degradation of allergenic epitopes compared to their undigested state (Figure 4B). Notably, FSPI digestate demonstrated a greater reduction in allergenic epitopes compared to SPI digestate, particularly in the G1, G2, and G4 subunits of glycinin, as well as the α and β subunits of β-conglycinin, alongside the basic 7S globulin. Three-dimensional structural diagrams revealed that the SPI digestate predominantly targeted the degradation of the external α-helix and irregular curl structures of the G1 subunit of glycinin, whereas the degradation of G2 and G4 subunits was primarily focused on the internal β-sheet structures (highlighted in blue, Figure 4B). The degradation of β-conglycinin and the basic 7S globulin by SPI was relatively complete, whereas the FSPI digestate further enhanced the degradation of epitopes in these proteins’ external regions (highlighted in green, Figure 4B). In comparison to FSPI digestates, samples that included added polysaccharides demonstrated higher degradation rates for allergenic epitopes. Specifically, the F-PDX digestate showed increased degradation rates for the G5 subunits of glycinin and the α and α’ subunits of β-conglycinin, with rates rising from 50.0%, 73.3%, and 70.5% in FSPI digestate to 56.2%, 76.7%, and 72.7% in F-PDX digestates. The 3D structural diagrams indicated that the further degraded allergenic epitopes were predominantly concentrated in the external α-helix structures of β-conglycinin (highlighted in red, Figure 4B). Notably, the glycinin G5 subunit exhibited a higher epitope degradation rate in F-PDX both before and after digestion compared to FSPI. This suggests that the addition of the polysaccharide PDX may facilitate LAB proteomic degradation, thereby enhancing the disruption of epitopes during gastrointestinal digestion. Furthermore, the degradation rates of the α and α’ subunits of β-conglycinin were comparable to FSPI before digestion, but were higher in the F-PDX digestates. This discrepancy may be attributed to PDX’s influence on the structure or arrangement of these allergens, ultimately aiding the enzymatic hydrolysis process and leading to the more extensive degradation of these subunits during digestion. The F-IN digestate showed increased degradation rates for the G1, G2, and G3 subunits of glycinin, as well as the basic 7S globulin, with percentages rising from 85.3%, 88.7%, 68.2%, and 71.0% in the FSPI digestate to 88.2%, 92.5%, 72.7%, and 84.2% in the F-IN digestate. Three-dimensional diagrams indicated that, compared to the FSPI digestate, the F-IN digestate significantly enhanced the degradation of the external α-helix structures of the basic 7S globulin (highlighted in yellow, Figure 4B). Notably, the glycinin G1 subunit exhibited a higher epitope degradation rate in F-IN than in FSPI, both before and after digestion. This observation suggests that the inclusion of polysaccharide IN enhances protein degradation by LAB, thereby improving the disruption of epitopes during gastrointestinal digestion. The degradation rates of the G2 and G3 subunits of glycinin and the basic 7S globulin were similar in FSPI before digestion, but showed higher rates in F-IN digestates after digestion. This may result from the integration of IN, which affects the structure or arrangement of these allergens, thus facilitating the enzymatic hydrolysis process and leading to the more extensive degradation of these subunits during digestion.

The addition of polysaccharides yielded different epitope disruption profiles for various allergens. Polysaccharides facilitated the degradation of certain allergens during fermentation, such as glycinin G5 in F-PDX and glycinin G1 in F-IN, which resulted in the greater breakdown of their primary structures and improved epitope degradation profiles in their respective digestates. In contrast, for other allergens, including the β-conglycinin α and α’ subunits in F-PDX, as well as glycinin G2, G3 subunits and the basic 7S globulin in F-IN, the addition of polysaccharides mainly promoted the epitope degradation rate after gastrointestinal digestion, which might be due to the interruption and rearrangement of protein higher-order structures, thereby improving their degradation profiles during digestion. Previous studies indicated that LAB fermentation significantly altered the higher structure of soy protein. Additionally, disruption of the primary structure occurred, including the α and β subunits of β-conglycinin and the G1 and G2 subunits of glycinin; some allergenic epitopes were degraded, thereby reducing the protein’s allergenicity [24]. Our study illustrates that the incorporation of polysaccharides PDX and IN into fermented soy protein may further enhance modifications to both primary and higher-order structures, as well as protein rearrangement. These changes ultimately influence the degradation rate during digestion, resulting in a reduction in protein allergenicity.

#### 3.4.2. Epitope Degradation Frequency of Major Soy Allergens

**Figure 5:**
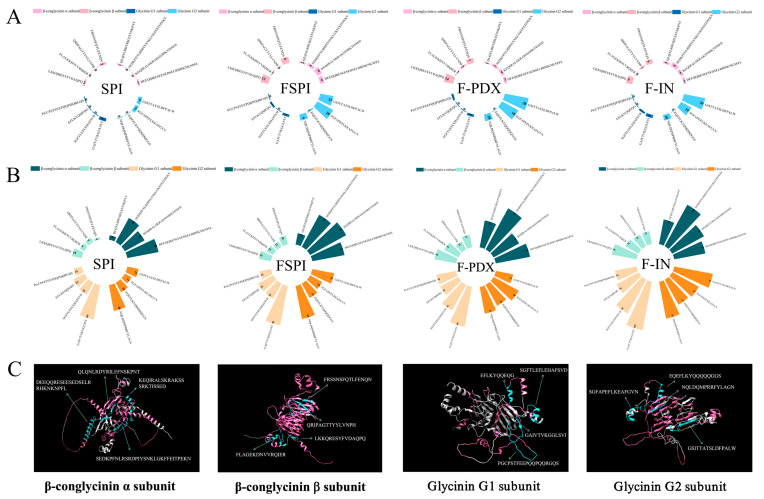
Degradation frequency of major allergen epitopes at I-0 (**A**) and I-120 (**B**) and major allergen epitopes in 3D models (**C**). (Allergenic epitopes are labeled with pink color, high frequency disrupted epitopes are marked with blue color).

Previous results indicated that different samples exhibited varying degradation rates in the glycinin G1 and G2 subunits, as well as in the β-conglycinin α and β subunits. Consequently, these subunits were selected for further investigation into epitope degradation frequency. Based on the peptidomic data, a total of sixteen peptides with the highest degradation frequencies were identified for analysis, which included four epitopes from each of the glycinin G1 subunit, glycinin G2 subunit, β-conglycinin α subunit, and β-conglycinin β subunit, as illustrated in Figure 5A,B. The degradation profiles and structural characteristics of the allergen epitopes are depicted using a 3D model in Figure 5C.

At I-0, the three FSPIs demonstrated higher degradation frequencies for certain epitopes (Figure 5A). The epitopes LKKQRESYFVDAQPQ (β-conglycinin β subunit), GSITTATSLDFPALW (glycinin G2 subunit), and SGFAPEFLKEAFGVN (glycinin G2 subunit) showed degradation frequencies of two, five, and five times, respectively, in SPI samples. In comparison, the frequencies were 12, 21, and 28 times in FSPI samples, 14, 38, and 18 times in F-PDX samples, and 9, 30, and 19 times in F-IN samples. In the 3D structural diagram, all three epitopes were positioned on the exterior of the protein structure, with LKKQRESYFVDAQPQ and SGFAPEFLKEAFGVN located within the α-helix structure, and GSITTATSLDFPALW situated in the β-sheet structure (Figure 5C).

After gastrointestinal digestion, all subunits demonstrated increased frequencies of allergenic epitope degradation, particularly the GAIVTVKGGLSVI and NQLDQMPRRFYLAGN epitopes found in the glycinin G1 and G2 subunits, respectively, with degradation frequencies exceeding 45 in all four samples (Figure 5B). Fermentation further enhanced the degradation of allergenic epitopes in the digestion products, especially in the samples containing added polysaccharides. For example, the epitopes SEDKPFNLRDPIYSNKLGKFFEITPEKN (β-conglycinin α subunit), LKKQRESYFVDAQPQ (β-conglycinin β subunit), SGFTLEFLEHAFSVD (glycinin G1 subunit), and EQEFLKYQQQQQGGS and SGFAPEFLKEAFGVN (glycinin G2 subunit) were degraded 98, 46, 61, 50, and 60 times, respectively, in the F-PDX digestate, and 104, 56, 85, 63, and 67 times in the F-IN digestate. These degradation rates were 1.3/1.4, 1.8/2.2, 1.7/2.4, 1.5/1.9, and 2.1/2.4 times higher than those observed in the FSPI digestate. Notably, most epitopes experienced more significant degradation in the F-IN digestate compared to the F-PDX digestate. The 3D structural analysis revealed that these epitopes are predominantly situated on the exterior of the protein structure, with two epitopes from β-conglycinin within the β-sheet structures and three epitopes from glycinin located in the α-helix structures (Figure 5C). This observation suggests that the addition of polysaccharides enhanced the degradation of β-sheet structures in the external regions of β-conglycinin and α-helix structures in the external regions of glycinin. Previous studies utilizing synthetic peptide techniques identified the SGFAPEFLKEAFGVN epitope of the glycinin G2 subunit as a significant IgE-binding site with high allergenicity [25]. Therefore, the substantial degradation of this epitope by F-PDX and F-IN may have contributed to its reduced allergenicity. This indicates that the softer and more fragmented network structure in F-PDX and F-IN allows digestive enzymes to hydrolyze allergenic epitopes more efficiently, leading to a faster reduction in allergenicity.

## 4. Conclusions

This study successfully developed a low-allergenic soy protein through fermentation with LAB, and the effects of polysaccharide supplementation on the structural characteristics and sensitization of the fermented soy protein gel were explored. Among all samples investigated, those supplemented with PDX and IN exhibited lower hardness, characterized by small, irregular pores and weaker honeycomb structures. In contrast, samples containing SSPS and BG displayed higher hardness, with larger, regular pores and robust sheet structures. The allergenicity results indicated that fermentation significantly reduces the antigenicity and IgE-binding capacity of soy proteins. When compared to SPI, the antigenicity of all FSPIs decreased to below 30%, whereas the IgE-binding capacity dropped to below 35%. Notably, samples that included the polysaccharides PDX and IN exhibited even lower allergenicity, with values ranging from 21.56% to 21.79%. The immunoreactivity of all fermented samples experienced a significant reduction during the later stages of digestion. Among these samples, the antigenicity and IgE-binding capacity of the products that underwent digestion from those containing polysaccharides PDX and IN were reduced to 0.0075% and 0.70%, respectively. Peptidomic analysis revealed that LAB fermentation promoted the degradation of allergenic epitopes in soy protein, with particular emphasis on glycinin and β-conglycinin. In comparison to FSPI, incorporating the polysaccharide PDX in the fermented samples further enhanced the degradation rate of the soybean globulin G5 subunit, both before and after digestion, whereas the incorporation of polysaccharide IN improved the degradation rate of the soybean globulin G1 subunit. The degraded epitopes were primarily concentrated within the external α-helical structures. In conclusion, the addition of polysaccharides to the SPI solution, followed by fermentation with LAB to form soy protein gels, significantly modifies the structural characteristics of the gels. These alterations subsequently influence the rate and extent of digestion in the stomach and duodenum, ultimately affecting their allergenic properties. In addition, polysaccharides may interact with proteins, causing changes in the secondary and tertiary structures of soy protein and modifying allergenic epitopes, thereby reducing allergenicity. This study systematically investigates, for the first time, the influence of various polysaccharides on the structure and allergenicity of fermented soy protein gels. These findings highlight the potential of using polysaccharides as additives to develop hypoallergenic soy products, making soy protein a safer food source for individuals with allergies and enhancing its nutritional acceptability and applicability.

## Figures and Tables

**Table 1:** Chemical characteristics of the different polysaccharides.

Polysaccharide	Monomeric Units	Chemical Linkages	Degree of Polymerization (DP)
PDX	Glucose, sorbitol, citric acid	Glycosidic bonds between glucose units	Typically ranges from 10 to 20 glucose units
IN	β-D-fructofuranose units	β(2→1) glycosidic bonds	2–60
LCIN	β-D-fructofuranose units	β(2→1) glycosidic bonds	>60
SSPS	Glucose units	β(1→4) glycosidic bonds	Varies, but typically short to medium length chains
BG	Glucose units	β(1→3) and β(1→4) glycosidic bonds	Varies, typically long-chain

**Table 2:** Textural properties of FSPI, F-PDX, F-IN, F-LCIN, F-SSPS, and F-BG.

Sample	Hardness (N)	Springiness (mm)	Cohesiveness (Ratio)	Gumminess (N)
FSPI	0.33 ± 0.01 abc	6.63 ± 0.05 d	0.52 ± 0.01 a	0.16 ± 0.00 ab
F-PDX	0.27 ± 0.03 c	6.97 ± 0.21 c	0.54 ± 0.02 a	0.14 ± 0.00 b
F-IN	0.28 ± 0.03 bc	7.30 ± 0.31 c	0.54 ± 0.02 a	0.19 ± 0.01 a
F-LCIN	0.36 ± 0.03 a	8.30 ± 0.07 a	0.52 ± 0.01 a	0.17 ± 0.01 a
F-SSPS	0.37 ± 0.07 a	7.83 ± 0.15 b	0.54 ± 0.02 a	0.18 ± 0.02 a
F-BG	0.39 ± 0.01 a	7.02 ± 0.27 c	0.54 ± 0.01 a	0.18 ± 0.02 a

Values after ± represent the standard deviations. a–d values followed by different letters in the same column are significantly different (*p* < 0.05).

## Data Availability

The data presented in this study are available on request from the corresponding author due to privacy considerations. However, interested researchers can request access to the data presented in this study by contacting the corresponding author. We will review and process data requests while ensuring privacy protection.

## Supplementary Materials

The following supporting information can be downloaded at: www.mdpi.com/article/10.3390/foods14040701/s1, Figure S1. Standard curve for antigenicity measurement; Table S1. Absorbance values of SPI, F-PDX, F-IN, F-LCIN, F-SSPS, and F-BG at 450 nm.

## References

[1] Michel F., Hartmann C., Siegrist M. (2021). Consumers’ Associations, Perceptions and Acceptance of Meat and Plant-Based Meat Alternatives. Food Qual. Prefer..

[2] Zhang T., Dou W., Zhang X., Zhao Y., Zhang Y., Jiang L., Sui X. (2021). The Development History and Recent Updates on Soy Protein-Based Meat Alternatives. Trends Food Sci. Technol..

[3] Wang J., He Z., Raghavan V. (2023). Soybean Allergy: Characteristics, Mechanisms, Detection and Its Reduction through Novel Food Processing Techniques. Crit. Rev. Food Sci. Nutr..

[4] Wiederstein M., Baumgartner S., Lauter K. (2023). Soybean (*Glycine max*) allergens—A Review on an Outstanding Plant Food with Allergenic Potential. ACS Food Sci. Technol..

[5] Luo J., Zhang Q., Gu Y., Wang J., Liu G., He T., Che H. (2022). Meta-Analysis: Prevalence of Food Allergy and Food Allergens—China, 2000–2021. China CDC Wkly..

[6] Tedner S.G., Asarnoj A., Thulin H., Westman M., Konradsen J.R., Nilsson C. (2022). Food Allergy and Hypersensitivity Reactions in Children and Adults—A Review. J. Intern. Med..

[7] Rui X., Fu Y., Zhang Q., Li W., Zare F., Chen X., Jiang M., Dong M. (2016). A Comparison Study of Bioaccessibility of Soy Protein Gel Induced by Magnesiumchloride, Glucono-δ-Lactone and Microbial Transglutaminase. LWT-Food Sci. Technol..

[8] Biscola V., De Olmos A.R., Choiset Y., Rabesona H., Garro M.S., Mozzi F., Chobert J.-M., Drouet M., Haertlé T., Franco B.D.G.M. (2017). Soymilk Fermentation by *Enterococcus faecalis* VB43 Leads to Reduction in the Immunoreactivity of Allergenic Proteins β-Conglycinin (7S) and Glycinin (11S). Benef. Microbes.

[9] Yang A., Zuo L., Cheng Y., Wu Z., Li X., Tong P., Chen H. (2018). Degradation of Major Allergens and Allergenicity Reduction of Soybean Meal through Solid-State Fermentation with Microorganisms. Food Funct..

[10] Liu Z., Fu Y., Liu Y., Chen X., Jiang M., Rui X. (2023). Lactic Acid Bacteria Fermented Soy β-Conglycinin: Assessment of Structural Conformational Feature and Immunoglobulin E Reactivity. LWT.

[11] Wu C., Wu F., Ju Q., Zhang Y., Yuan Y., Kang S., Hu Y., Luan G. (2023). The Role of β-Subunit in Emulsifying Performance of β-Conglycinin. Food Hydrocoll..

[12] Yang X., Ren Y., Liu H., Huo C., Li L. (2021). Differences in the Physicochemical, Digestion and Microstructural Characteristics of Soy Protein Gel Acidified with Lactic Acid Bacteria, Glucono-δ-Lactone and Organic Acid. Int. J. Biol. Macromol..

[13] Jayasree Subhash A., Babatunde Bamigbade G., al-Ramadi B., Kamal-Eldin A., Gan R.-Y., Senaka Ranadheera C., Ayyash M. (2024). Characterizing Date Seed Polysaccharides: A Comprehensive Study on Extraction, Biological Activities, Prebiotic Potential, Gut Microbiota Modulation, and Rheology Using Microwave-Assisted Deep Eutectic Solvent. Food Chem..

[14] Zhao H., Wang S., Zhao G., Li Y., Liu X., Yang L., Zhu L., Liu H. (2022). Fabrication and Emulsifying Properties of Non-Covalent Complexes between Soy Protein Isolate Fibrils and Soy Soluble Polysaccharides. Food Funct..

[15] Guggisberg D., Cuthbert-Steven J., Piccinali P., Bütikofer U., Eberhard P. (2009). Rheological, Microstructural and Sensory Characterization of Low-Fat and Whole Milk Set Yoghurt as Influenced by Inulin Addition. Int. Dairy J..

[16] L’hocine L., Boye J.I., Arcand Y. (2006). Composition and Functional Properties of Soy Protein Isolates Prepared Using Alternative Defatting and Extraction Procedures. J. Food Sci..

[17] Minekus M., Alminger M., Alvito P., Ballance S., Bohn T., Bourlieu C., Carrière F., Boutrou R., Corredig M., Dupont D. (2014). A Standardised Static In Vitro Digestion Method Suitable for Food—An International Consensus. Food Funct..

[18] Bai J., Zeng Q., Den W., Huang L., Wu Z., Li X., Tong P., Chen H., Yang A. (2025). Synergistic Synbiotic-Containing Lactiplantibacillus Plantarum and Fructo-Oligosaccharide Alleviate the Allergenicity of Mice Induced by Soy Protein. Foods.

[19] Pi X., Liu J., Sun Y., Ban Q., Cheng J., Guo M. (2023). Protein Modification, IgE Binding Capacity, and Functional Properties of Soybean Protein upon Conjugation with Polyphenols. Food Chem..

[20] Dos Santos A.L.S., Dos Santos P.P.B., De Almeida Amaral G., Soares E.C., De Oliveira E Silva C.A., De Souza S.V.C. (2022). Effect of Thermal Processing on the Antigenicity of Allergenic Milk, Egg and Soy Proteins. J. Food Sci. Technol..

[21] Han C., Yang X., Li L. (2023). Physicochemical Properties and Microstructure of Soybean Protein Isolate-Vegetable Oil Complex Gels Induced by Lactic Acid Bacteria: Effects of Vegetable Oil Types and Concentrations. Food Hydrocoll..

[22] Wang Y., Fu Y., Azarpazhooh E., Li W., Liu Q., Rui X. (2022). Assessment of in Vitro Digestive Behavior of Lactic-Acid-Bacteria Fermented Soy Proteins: A Study Comparing Colloidal Solutions and Curds. Molecules.

[23] Yekta R., Assadpour E., Hosseini H., Jafari S.M. (2023). The Influence of Ionic Polysaccharides on the Physicochemical and Techno-Functional Properties of Soy Proteins; a Comprehensive Review. Carbohydr. Polym..

[24] Lu Q., Zuo L., Wu Z., Li X., Tong P., Wu Y., Fan Q., Chen H., Yang A. (2022). Characterization of the Protein Structure of Soymilk Fermented by Lactobacillus and Evaluation of Its Potential Allergenicity Based on the Sensitized-Cell Model. Food Chem..

[25] Xiang P., Beardslee T.A., Zeece M.G., Markwell J., Sarath G. (2002). Identification and Analysis of a Conserved Immunoglobulin E-Binding Epitope in Soybean G1a and G2a and Peanut Ara h 3 Glycinins. Arch. Biochem. Biophys..

