# Supplementary material for: Effects of Polysaccharide Supplementation on Lactic Acid Bacteria-Fermented Soy Protein Gel: Structural Characteristics, Allergenicity, and Epitope Analysis
Source: Foods. 2025 Feb 18;14(4):701. doi: 10.3390/foods14040701 (PMC11854590; doi:10.3390/foods14040701)
Supplement: Supplementary file 1 [file foods-14-00701-s001.zip › foods-3442590-supplementary.pdf]

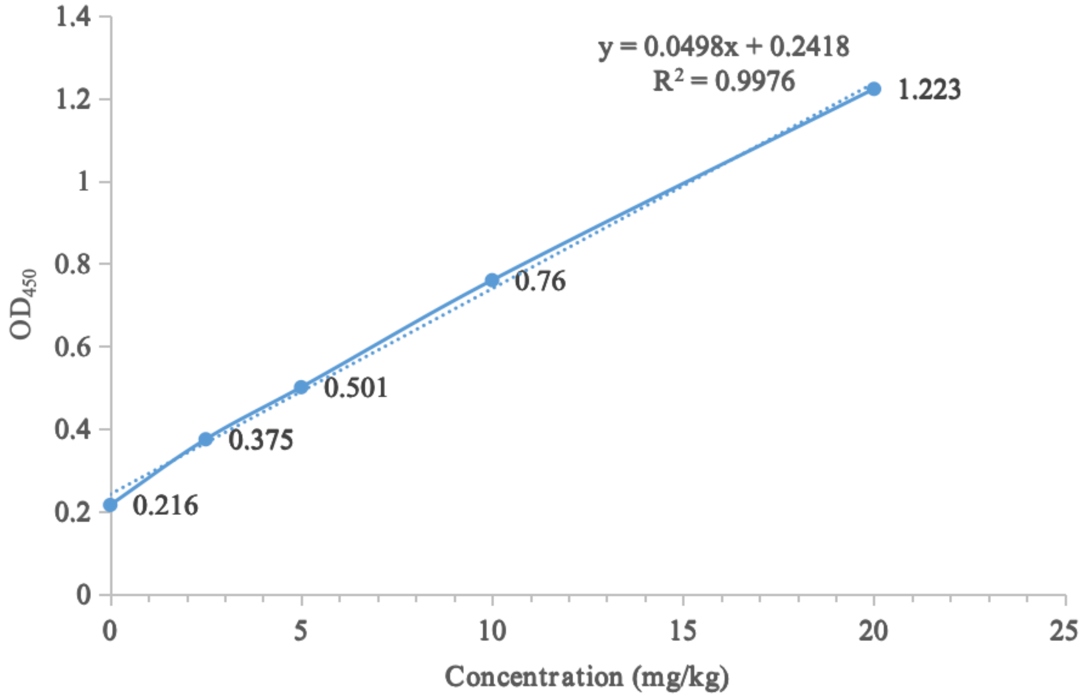

Figure S1. Standard curve for antigenicity measurement

Table S1. Absorbance values of SPI, F-PDX, F-IN, F-LCIN, F-SSPS, and F-BG at 450 nm

Dilution ratio (times)	Digestion time	SPI	FSPI	F-PDX	F-IN	F-LCIN	F-SSPS	F-BG
		0.741	0.385	0.352	0.349	0.38	0.363	0.385
35000	I-0	0.794	0.387	0.355	0.354	0.387	0.361	0.38
		0.756	0.382	0.351	0.355	0.382	0.37	0.374
		0.892	0.403	0.362	0.355	0.423	0.387	0.39
50	I-60	0.921	0.376	0.36	0.368	0.444	0.38	0.402
		0.935	0.389	0.359	0.36	0.445	0.381	0.395
		0.356	0.356	0.289	0.291	0.305	0.352	0.297
50	I-120	0.377	0.349	0.25	0.283	0.263	0.349	0.29
		0.36	0.34	0.267	0.267	0.298	0.35	0.295
		0.314	0.304	0.247	0.279	0.272	0.314	0.274
50	I-180	0.328	0.301	0.243	0.27	0.261	0.304	0.278
		0.318	0.278	0.245	0.272	0.27	0.31	0.276
